# Identifying gene-environment interactions across genome-wide, twin, and polygenic risk score approaches

**DOI:** 10.3389/fgene.2025.1698381

**Published:** 2025-11-07

**Authors:** Brad Verhulst

**Affiliations:** Department of Psychiatry and Behavioral Sciences, Texas A&M University, College Station, TX, United States

**Keywords:** gene-environment interaction (GxE), twin models, polygenic risk score (PRS), genome-wide association study (GWAS), data simulation

## Abstract

**Introduction:**

Until recently, many researchers have been hesitant to conduct genome-wide gene-environment interaction (GxE) research due to perceptions of low rates of statistical power and skepticism from controversial findings from the existing literature. Nevertheless, twin and polygenic risk score (PRS) studies suggest that GxE is pervasive and may have a large impact on complex genetic traits. Our goal in this paper is to demonstrate that consistent findings emerge from twin, PRS, and genome-wide approaches to identify GxE, subject to the known limitations for each method.

**Method:**

We conducted a series of simulation studies, generating dataset that can be used in twin, PRS and GWAS analyses.

**Results:**

We highlight a high degree of consistency across approaches, with each method detecting GxE. Specifically, genome-wide approaches identify individual variants that interact with an environmental moderator, but struggle with low statistical power when a trait is highly polygenic. Alternatively, aggregating genome-wide effects from a discovery sample into a PRS in the target sample increases the ability to detect broad genetic effects. However, if the statistical power in the discovery sample is low, the associations with the PRS tend to underestimate the genetic signal. This is true for both genetic main and interaction effects. Finally, twin studies are generally robust to differences in polygenicity as well as the underlying distributions of the genetic main and interaction effects. The ability of all three methods to robustly identify genomic moderation emphasizes the fact that multiple valid ways to detect GxE exist that stem from the same basic assumptions about the genetic architecture of complex traits.

## 1 Introduction

Genetic associations often depend on contextual factors that amplify or suppress the relationships between genotypes and phenotypes. These interactions are broadly referred to as gene-environment interaction (or GxE). Genome-wide association study (GWAS) findings emphasize the incredible complexity of the relationships between genetic factors and almost every aspect of human behavior and disease. However, traditional GWAS approaches ignore GxE and the mechanisms through which contextual factors potentially regulate phenotypic outcomes (Verhulst et al., 2021). Identifying the genetic variants that respond to contextual variation may provide critical insights into behavior modification. The majority of GxE evidence comes from twin studies, polygenic risk score (PRS) by environment interaction (PRSxE) studies, and candidate gene-environment interaction studies (which we do not discuss in detail; see Duncan and Keller, 2011). Genome-wide GxE approaches remain relatively unexplored, creating the artificial perception of methodological barriers for understanding genomic sensitivity to contextual differences. While each method has unique strengths and limitations, our overarching premise is that if GxE exists, we should be able to detect it with multiple methods, *ceteris paribus*. In this study, we present a series of simulation studies to illustrate the interconnected analytical underpinnings between twin, PRS, and genome-wide GxE models. The simulation ensures that the same data, or portions thereof, can be used for all analyses. The accompanying R script can be found on GitHub (https://github.com/bradverhulst/GxESimulation/), where readers can reproduce the analyses and extend them to explore nuanced variations of GxE in different scenarios.

The simulation studies we present steadily increase in complexity, from a single variant with no moderation to a highly polygenic system with extensive moderation. Despite the complexity of these simulations, real world analyses are substantially more complex. Accordingly, the simulation studies highlight potential mathematical and biological processes that could affect the statistical decision making in GxE studies, as well as problems that may arise in the interpretation of the results. The major benefit of simulation studies is that we know what the results should look like, and therefore, are able to identify how each methodological approach is affected by the data generating process.

### 1.1 Defining “genes” and “environments”

Biologically, a gene is a sequence of nucleotides that contain the necessary information for making a protein that contributes to the expression of one or more physical characteristics or traits (Gerstein et al., 2007). However different methods operationalize “genes” as a variety of interrelated constructs focusing on inherited biological differences. For example, in twin and family models, genes imply a latent biological predisposition for a behavior (Neale and Cardon, 1992). PRS analyses, by contrast, conceptualize genes as an aggregated or weighted sum of the relevant risk alleles for a behavior across the genome (Cross et al., 2022). Alternatively, GWASs focus on single nucleotide polymorphisms (SNPs), or small insertions or deletions (indels), and have a tendency of blurring the distinction between SNPs/indels and genes. Each of these conceptualizations of genes can interact with environmental factors to amplify or suppress the likelihood of a behavior. The implications of these distinct definitions of genes for GxE is heterogeneous, as different operationalizations of genes constrain how environments may moderate their association with a phenotype.

Defining the environment is a similarly amorphous task. In the broadest sense, an environment is anything that is not a gene. The extreme breadth of this definition may include literal environments such as the proximity to parks and greenspaces (Reed et al., 2022), inferred environments such as socioeconomic status (Abdellaoui et al., 2025), adverse events like physical assault or maltreatment (Morrison et al., 2021), or even Petri dishes with different types of agars. More restrictive definitions require that environments are not heritable or influenced by genetic factors. However, such narrow definitions are complicated by the fact that genetic factors often cryptically contribute to variables that are typically considered environments (Kendler and Baker, 2007; McAdams et al., 2013), such as where a person lives, their social networks, and other social determinants of health and behavior. Accordingly, almost no environment satisfies the strict definition. From a GxE perspective, the fact that moderators may have a genetic component complicates the interpretation of any findings, making it difficult, if not impossible, to disaggregate pure gene-environment interactions from gene-gene interactions, or gene-moderator interactions. To circumvent this potential quagmire, when we refer to GxE, we focus on gene-moderator interactions, accepting any potential interpretational ambiguity that may be implied.

## 2 Methods

**FIGURE 1:**
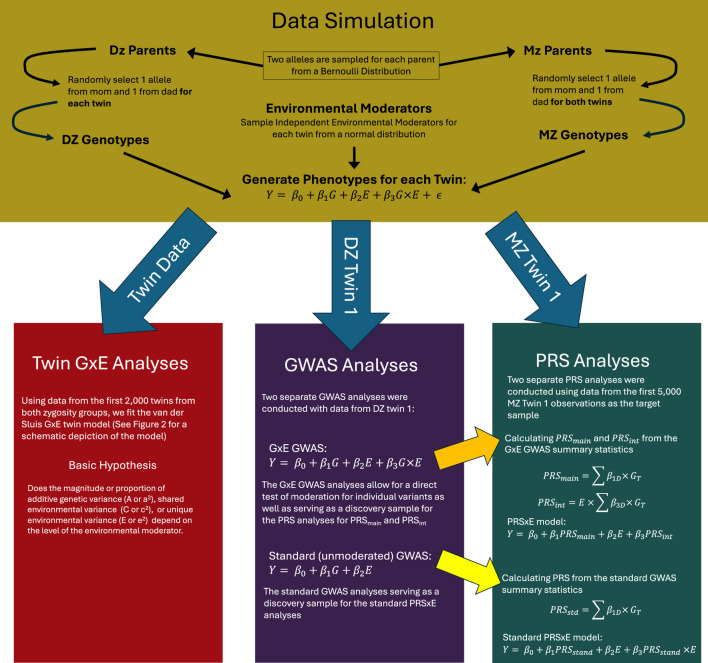
A schematic overview of the data simulation and analysis procedures.

A series of simulation studies were conducted to examine the ability of GWAS, PRS and twin models to detect GxE. All simulations were conducted in R (v4.5.1; R Core Team, 2025) using MASS 7.3 (Venables and Ripley, 2002) for generating data. Twin analyses were conducted with OpenMx 2.22.7 (Boker et al., 2011; Neale et al., 2016). All other analyses were conducted using available functions within R. Several functions were defined to simplify the analyses, which can be found on GitHub (https://github.com/bradverhulst/GxESimulation/). All the code to simulate data, conduct the analyses, and plot the results are also available on GitHub. A schematic overview of the data simulation and analysis procedures is presented in Figure 1.

### 2.1 Data simulation procedures

Data were generated so that the same data could be simultaneously used to conduct twin, PRS and GWAS analyses. In the data simulation, we generated genotypes for 100,000 DZ mothers and fathers (and 100,000 MZ parental dyads) by randomly sampling each allele from a binomial distribution: two alleles (A1 and A2) for each parent. The maternal and paternal alleles were then summed to obtain the respective genotypes. Random normal variables were then generated to approximate the residual environmental variance for each parent. To generate the DZ genotypes, we randomly sampled one maternal and one paternal allele for each twin, which produces a correlation of r = 0.50 between the genotypes across the DZ twin pairs. For the MZ genotypes, we sampled one maternal and one paternal allele for both twins, which produces a correlation of r = 1 between the MZ genotypes. For each twin type, values for the moderators and residual variances were sampled from a normal distribution.

Continuous, quantitative phenotypes for each twin were constructed based on the algebra for the moderated regression formula (Equation 1). Regression coefficients for the direct effect of the genotypes on the phenotypes and the interaction effects were generated under two conditions. For Studies 1-3, regression coefficients had a constant effect where the sum of the squared genetic effects was 1. In Studies 2 and 3, the sum of the squared moderated effects was also 1. For Study 4, regression coefficients were drawn from a normal distribution with a mean of zero and a variance of ${\left(1/nSNPs\right)}^{2}$ . Interested users can use the data generating functions from GitHub to specify the magnitude of the direct effects of the moderator and the intercept, but this is not discussed.

### 2.2 Twin models

**FIGURE 2:**
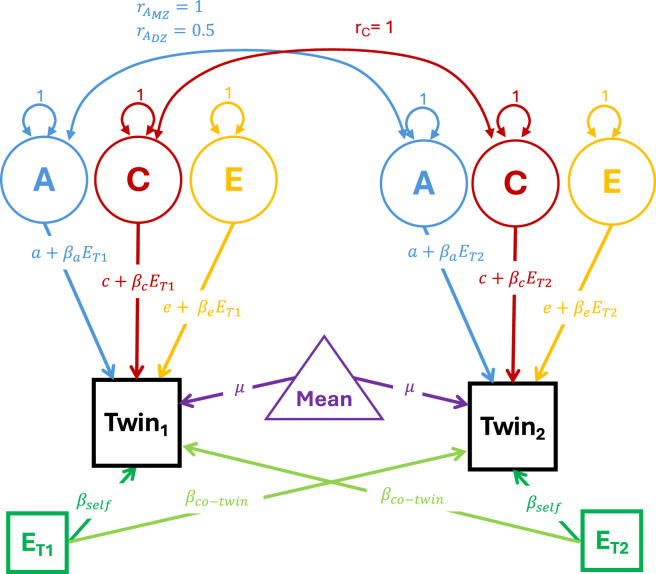
Path diagram of the Van Der Sluis et al. (2012) GxE model. Note: The phenotypic variance in the phenotypes for twins 1 and 2 is decomposed into additive genetic (A), shared environmental (C) and unique environmental E variances. Consistent with standard structural equation modeling conventions, circles indicate latent variables, squares indicate observed variables and triangles indicate means or constants. Double headed arrows indicate covariances while single headed arrows indicate regression paths. Paths with numbers (such as the variances of the latent variance components) indicate that the parameter is fixed to that value during optimization. Paths with equations or parameters indicate the estimated associations between the variables.

Twin GxE models were fit using structural equation modeling in OpenMx (Neale et al., 2016). In standard twin models, phenotypic variance is decomposed into additive genetic variance (Va or a^2^), common environmental variance (Vc or c^2^), and unique environmental variance (Ve or e^2^). Twin GxE models extend this framework by adding a moderation parameter to each variance component that allows for the amplification or suppression of that specific source of variance: i.e., (a + $\beta_{a}$ mod)^2^, (c + $\beta_{c}$ mod)^2^, and (e + $\beta_{e}$ mod)^2^. The twin GxE models conducted here rely on the van der Sluis specification (Van Der Sluis et al., 2012). A path diagram of the van der Sluis GxE twin model is presented in Figure 2. In the van der Sluis GxE model, in addition to adding moderation to the A, C and E paths, both twin’s phenotypes are regressed on their moderating environment as well as their co-twin’s moderator to reduce the influence of gene-environment correlation on the moderation parameters. This model is more robust to parameter bias in situations where gene-environment correlation is present than comparable models. To ensure that the same data was used in all analyses, we simulated data for 100,000 MZ and 100,000 DZ twin pairs. For a twin model, this sample size is beyond ridiculous. Extremely large twin registries have between 6,000 and 8,000 twin families (Lake et al., 2000; Truett et al., 1994), but other twin studies use data from about 1,000 twin families. Thus, to ensure our results are broadly consistent with the existing literature, rather than using the entire simulated sample, for all twin analyses we selected the first 2000 MZ and 2000 DZ twin pairs.

### 2.3 GWAS models

The GxE GWAS analyses uses the simulated data from DZ twin 1 in a standard moderated regression framework to sequentially regress the phenotype on each SNP in the genome, a moderator, and the interaction between each SNP and the moderator, as presented in Equation 1.

$$Y_{i}={\hat{\beta }}_{0j}+{\hat{\beta }}_{1j}{SNP}_{ij}+{\hat{\beta }}_{2j}{Env}_{i}+{\hat{\beta }}_{3j}{SNP}_{ij}{Env}_{i}+\varepsilon_{ij}$$ (1)

where $Y_{i}$ is a continuous phenotype and ${Env}_{i}$ is a binary environmental moderator for the *ith* person, and ${SNP}_{ij}$ is the *jth* SNP for the *ith* person. Accordingly, for the *jth* SNP, ${\hat{\beta }}_{0j}$ intercept, ${\hat{\beta }}_{1j}$ is the main or additive SNP effect, ${\hat{\beta }}_{2j}$ is the environmental moderator effect, and ${\hat{\beta }}_{3j}$ is the interaction effect. The $\varepsilon_{ij}$ parameter captures residual variation for the *jth* SNP.

In addition to being of interest in their own rights, the ${\hat{\beta }}_{1j}$ and ${\hat{\beta }}_{3j}$ summary statistics from the GxE GWAS of DZ twin 1 are used as the input for the PRSxE analyses (described below).

For standard (unmoderated) GWASs, the outcome was regressed on the intercept, the environmental moderator, and each SNP in the simulated genome sequentially, as presented in Equation 2.

$$Y_{i}={\hat{\beta }}_{0j}+{\hat{\beta }}_{1j}{SNP}_{ij}+{\hat{\beta }}_{2j}{Env}_{i}+\varepsilon_{ij}$$ (2)

where $Y_{i}$ , ${Env}_{i}$ , ${SNP}_{ij}$ , and $\varepsilon_{ij}$ are defined as above, and ${\hat{\beta }}_{0j}$ is the intercept, ${\hat{\beta }}_{1j}$ is the main or additive SNP effect and ${\hat{\beta }}_{2j}$ is the environmental moderator effect estimated without the interaction effect.

All GWAS significance tests were assessed using Wald tests ( Z=β/se ). Multiple testing corrections were based on genome-wide significance (p < 5e-8) and Bonferroni corrections ( $\left.\alpha =0.05/nSNPs\right)$ . As we used simulated data, ancestry principal components and other stanard covariates were not generated but should undoubtedly be included in any analyses of real data. GWASs are inherently low powered analyses, with myriad SNPs affecting the phenotype, each with a small effect size (Visscher et al., 2014). As such, the most effective and uncontroversial way of boosting power is to increase sample size. In simulation studies, this is trivially simple, limited primarily by computer memory constraints. Accordingly, we simulated GWAS data from 100,000 MZ and DZ twin families, but to simplify the statistical assumptions (specifically the independence of observations assumption), we only analyze GWAS data for DZ twin 1. These analyses become the discovery analyses for the PRSxE analyses.

### 2.4 PRSxE models

As PRSs require that individuals in the discovery sample are unrelated to those in the target sample, we use GWAS analyses for DZ twin 1 as the discovery dataset, and the data for the first 5,000 MZ twin 1s as the target dataset. Because statistical power in PRSs studies comes disproportionately from the sample size of the discovery sample rather than the sample size of the target sample, using 5,000 observations in the target dataset is more appropriate than the full 100,000 observation sample. Intuitively, if the regression weights are estimated more accurately in the discovery sample, the PRS values in the target sample will be more reliable and precise (Dudbridge, 2013; Palla and Dudbridge, 2015). This enhanced reliability often corresponds with larger PRS effect sizes, smaller p-values, and larger *r* ^2^ values. While these observations have been made in the context of linear PRS analyses, they should equally apply to PRSxE analyses.

Across all the simulation studies, we conducted two separate PRSxE analyses using 1) the GxE GWAS and 2) the standard (unmoderated) GWAS summary statistics as the discovery analyses. These analyses closely follow Jayasinghe et al. (2024), Werme et al. (2021), and (Choi et al., 2020). Specifically, for the PRS analyses using the GxE GWAS summary statistics as the discovery sample, we constructed PRS_main_ from the main effect parameters ( ${\hat{\beta }}_{1Dj}$ ) and PRS_int_ the interaction effect parameters ( ${\hat{\beta }}_{3Dj}$ ). Specifically:

$${PRS}_{main}=\sum {\hat{\beta }}_{1Dj}{SNP}_{ij}$$ (3)

$${PRS}_{\mathrm{int}}=\mathrm{mod}\sum {\hat{\beta }}_{3Dj}{SNP}_{ij}$$ (4)

Where ${\hat{\beta }}_{1Dj}$ and ${\hat{\beta }}_{1Dj}$ are the regression weights for the *jth* SNP in the GxE GWAS discovery analysis, and ${SNP}_{ij}$ is the corresponding variant in the target sample.

The PRS analyses in the target dataset, regress the outcome ( $Y_{i}$ ) on PRS_main_, the environmental moderator, and PRS_int_:

$$Y_{i}={\hat{\beta }}_{0}+{\hat{\beta }}_{1}{PRS}_{main}+{\hat{\beta }}_{2}{Env}_{i}+{\hat{\beta }}_{3}{PRS}_{\mathrm{int}}$$ (5)

For the PRS analyses using the unmoderated GWAS summary statistics as the discovery sample, we constructed PRS_stand_ from the main effect parameters ( ${\hat{\beta }}_{1}$ ). Specifically:

$${PRS}_{stand}=\sum {\hat{\beta }}_{1Dj}{SNP}_{ij}$$ (6)

Where ${\hat{\beta }}_{1Dj}$ is the regression weight for the *jth* SNP in the standard GWAS discovery analysis. The interaction effect, then, is the product between PRS_stand_ and the environmental moderator. The standard PRSxE analyses are conducted using the model:

$$Y_{i}={\hat{\beta }}_{0}+{\hat{\beta }}_{1}{PRS}_{stand}+{\hat{\beta }}_{2}{Env}_{i}+{\hat{\beta }}_{3}{PRS}_{stand}{Env}_{i}$$ (7)

### 2.5 Simulation study parameters

Four simulations studies were conducted to examine how different levels of polygenicity, as well as the magnitude and distribution of the effect sizes influence the ability to identify GxE. To establish baseline expectations, study 1 examines a single variant with no interaction, where a one-allele increase in a single variant increases the phenotype by 1 unit, with a residual variance of 0.50. Study 2 extends the simulation to include GxE by adding a binary (yes/no) moderator that amplifies the effect of the single variant on the phenotype by an additional 1 unit for each additional allele if the moderator is “yes”, keeping the rest of the simulated parameters the same. Study 3 further expands the simulation, generating 1,000 independent SNPs that predict the phenotype, with a uniform effect size of β = $\sqrt{1/1000}$ = 0.0316 for each SNP. Further, we assume these SNPs interact with a binary environment at the same magnitude as the main effects. Under these conditions, the total genetic variation and moderation of the polygenic simulation mirror those of the single gene interaction analyses presented in study 2. Finally, in study 4 we conduct a more realistic simulation by simulating the distribution of the main effect and interaction coefficients from a normal distribution, allowing all variants to have a potential main effect and/or interaction effect. To maintain consistency with the previous simulations, the standard deviation of the normal distribution for the main and interaction effects was also set to $\sqrt{1/1000}$ so that the total genetic and interaction variance was approximately 1.

## 3 Results

### 3.1 Study 1: single variant without an interaction

**FIGURE 3:**
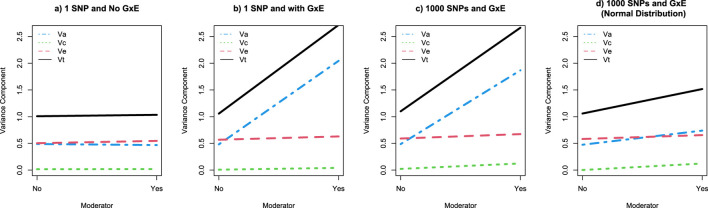
Graphical depiction of the twin GxE variance decomposition as a function of a moderating variable across 4 simulation studies. Note: Panels **(a–d)** present the variance decomposition results for simulations studies 1-4, respectively. Va is the variance accounted for by additive genetic factors and depicted by the blue dotted line; Vc is the variance accounted for by shared environmental factors and depicted by the green dotted line; Ve is the variance accounted for by unique environmental factors and depicted by the red dotted line; and Vt is the total variance (i.e., Va + Vc + Ve) depicted by the black line.

To begin, we generated genotypes for 100,000 MZ and DZ twin families (N_total_ = 800,000). As a proof of principle for the consistency across twin and genomic methods, we regressed each person’s genotype on their phenotype. Consistent with the data simulation procedures, the regression coefficient for the genetic association with the phenotype is approximately 1 and the *r* ^2^ is approximately 0.5 (Table 1). While there is only one variant in this analysis, it nevertheless relies on the standard GWAS framework. If we fit a standard ACE twin model to data from 2000 MZ and 2000 MZ twins, we get estimates of a^2^ = 0.5, c^2^ = 0, and e^2^ = 0.5, as shown in Figure 3a. As predicted from the biometrical model, the *r* ^2^ from the regression analyses is approximately the same as the a^2^ from the twin model.

**TABLE 1:** Estimated regression coefficients and *r* ^2^ statistics for parents and twins with data simulated with a single variant and no interaction.

	DZ twins				MZ twins			
	Twin 1	Twin 2	Mom	Dad	Twin 1	Twin 2	Mom	Dad
Intercept	0.00	0.00	0.00	−0.01	0.00	0.00	0.00	0.00
Beta_SNP_	1.00	1.00	1.00	1.00	1.00	1.00	1.00	1.00
*r* ^2^	0.50	0.50	0.50	0.50	0.50	0.50	0.50	0.50

The results present estimates for a simulated quantitative phenotype regressed on a simulated genetic variant for 100,000 parents and twins from MZ, and DZ, families (i.e., 800,000 individuals). Data were simulated so that a one-allele increase in a single genomic variant increased the phenotype by 1 unit, with a residual variance of 0.50 for everyone. Statistical significance measures were omitted from the table as the intercepts were all non-significant (p > 0.05) and the SNP, regression coefficients were all highly significant (p < 1e-16).

The PRS results for this data are presented in the left panels of Table 2. Because data was simulated without an interaction, in the PRSxE analyses using summary statistics from the GxE GWAS discovery models, PRS_main_ has the expected impact on the outcome and PRS_int_ has no effect. The PRSxE analyses from the standard GWAS discovery models recapitulate these findings: PRS_stand_ captures the genetic effects and the interaction effect is negligible. Furthermore, in both the GxE GWAS and standard GWAS discovery analyses, the *r* ^2^ is approximately 0.5 for both models. These results demonstrate that in the absence of GxE standard GWAS, PRS, and twin methods converge on the conclusion that genetic factors (in this case a single variant) account for ½ of the phenotypic variance.

**TABLE 2:** Results for the PRSxE analyses across the four simulation studies.

		Study 1	Study 2	Study 3	Study 4
		Estimate (SE)	Estimate (SE)	Estimate (SE)	Estimate (SE)
GxE GWAS Discovery Sample	Intercept	−0.046 (0.018)^*^	−0.021(0.024)	−1.004 (0.598)	−0.100 (0.016)^***^
	PRS_main_	1.031 (0.014)^***^	1.030 (0.020)^**^	1.048 (0.019)^***^	0.988 (0.014)^***^
	Moderator	−0.023 (0.018)	0.017 (0.035)	3.733 (0.805)^***^	0.077 (0.022)^***^
	PRS_int_	−2.307 (2.000)	0.974 (0.028)^***^	0.87 (0.025)^***^	0.964 (0.019)^***^
	*r* ^2^	0.505	0.703	0.998	0.722
Standard GWAS Discovery Sample
	Intercept	−0.046 (0.018)^*^	−0.021 (0.024)	1.387 (0.666)	−0.105 (0.021)^***^
	PRS_stand_	1.031 (0.014)^***^	1.030 (0.020)^***^	0.972 (0.021)^***^	0.978 (0.025)^***^
	Moderator	−0.023 (0.018)	0.017 (0.035)	0.794 (0.944)	0.449 (0.030)^***^
	PRS_stand_ x moderator	0.017 (0.014)	0.976 (0.029)^***^	0.991 (0.030)^***^	−0.022 (0.035)
	*r* ^2^	0.505	0.743	0.998	0.400

Study 1 consisted of a single variant with no GxE. Study 2 had a single variant with GxE. Study 3 had 1,000 variants all with GxE. Study 4 sampled main effects and interaction effects from independent normal distributions (with a mean effect size of zero). In the GxE GWAS discovery models, PRS_main_ refers to the PRSs derived from the ${\hat{\beta }}_{1}$ coefficient and PRS_int_ refers to the PRSs derived from the ${\hat{\beta }}_{3}$ from the moderated regression model presented in Equations 1, 3 and 4. The regression model for PRSs derived from the GxE discovery analyses is presented in Equation 5. For the Standard GWAS discovery model, PRS_stand_ refers to the PRSs derived from ${\hat{\beta }}_{1}$ from the standard GWAS presented in Equations 2 and 6, and evaluated via the regression model presented in Equation 7.

^*^ p < .05.

^**^ p < .01.

^***^ p < .001.

### 3.2 Study 2: single variant with an interaction

We present the moderated regression analyses for a single variant and an interaction for 400,000 twins (separated into MZ and DZ twins 1 and 2). These analyses are analogous to a GxE GWAS for a single variant. As can be seen in Table 3, the regression coefficients for each twin are recovered accurately, with the main and interaction effects both having regression coefficients of approximately 1, and the intercepts and moderator effects having virtually no effect. Interestingly, the interpretation of the *r* ^2^ values for the full model diverge from the variance accounted for in the moderator stratified analyses. Specifically, while the *r* ^2^ for the full model is approximately 0.75, when the environment is “no” the *r* ^2^ is 0.50, while the *r* ^2^ when the environment is “yes” is 0.80. Thus, the *r* ^2^ for the full model does not reflect the *r* ^2^ for either the yes or the no conditions. This is an important theme in moderation analyses, as *r* ^2^ may not be as instructive as it is in other linear regression, GWAS, or standard PRS analyses. The twin analyses, focusing on the first 2000 MZ and DZ twins, reiterate these observations. Plotting the variance components from the twin GxE model (Figure 3b) illustrates that the additive genetic variance component (Va) accounts for approximately half of the phenotypic variance (Va ≈ 0.5, Total variance or Vt ≈ 1) in the absence of the moderator, but around 80% of the phenotypic variance (Va ≈ 2, Vt ≈ 2.5) in the presence of the moderator.

**TABLE 3:** Moderated regression estimates and r^2^ statistics for each twin in the simulation study for a single variant with an interaction.

	DZ twins		MZ twins	
	Twin 1	Twin 2	Twin 1	Twin 2
Intercept	0.00	0.00	0.01	0.00
SNP	1.00	1.00	1.00	1.00
Moderator	−0.01	0.00	−0.01	0.00
SNP x Moderator	1.00	1.00	1.01	1.00
*r* ^2^ (Full model)	0.75	0.75	0.75	0.75
*r* ^2^ (Mod = No)	0.50	0.50	0.50	0.50
*r* ^2^ (Mod = Yes)	0.80	0.80	0.80	0.80

The results present moderated regression estimates for simulated quantitative phenotypes on a simulated genetic variant for 100,000 MZ, and DZ, twins (i.e., 400,000 individuals across 4 analyses). Data were simulated so that a one-allele increase in a single genomic variant increased the phenotype by 1 unit if the moderator was 0 (“No”), and by an additional 1 unit when the moderator is 1 (“Yes”), with a residual variance of 0.50 for everyone. Statistical significance indices were omitted as the intercepts and moderator main effects were all non-significant (p > 0.05) and the SNP, regression and interaction coefficients were all highly significant (p < 1e-16).

We present the results of the PRSxE analyses from the GxE discovery model and the standard GWAS discovery model in the second panels of Table 2. In this simplified case, the standard GWAS discovery sample results mirror the GxE GWAS discovery analyses results, with the parameter estimates being virtually identical in both models. As with the moderated regression analyses, the main and moderation effects are approximately 1, and the intercept and the moderator effects are approximately 0. The only subtle difference between the models is that the *r* ^2^ is larger in the standard GWAS discovery sample.

### 3.3 Study 3: polygenic model with constant main effects and interactions

**FIGURE 4:**
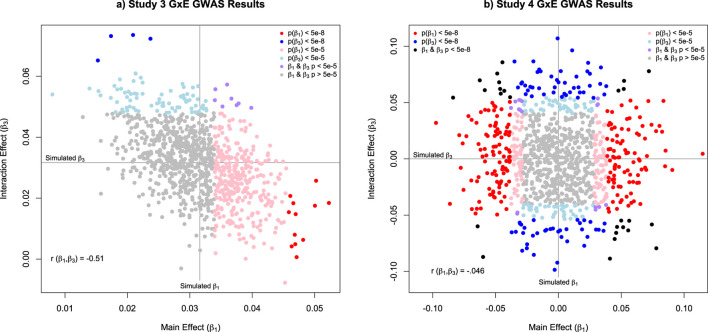
Scatterplot of the estimated main effect (β_1_) and interaction effect (β_3_) parameters from Studies 3 and 4. Note: The results for Study 3 are presented in panel **(a)**. For Study 3, the simulated values for all variants for both β_1_ and β_3_ were set to = 0.0316 to ensure that the total expected main and interaction effect were 1. The results for Study 4 are presented in panel **(b)**. For Study 4, the simulated values for β_1_ and β_3_ were drawn from two independent normal distributions with a standard deviation of = 0.0316, so that the total expected main and interaction effect variances were approximately 1.For both panels, the red and blue dots indicate variants that were genome-wide significant (p < 5e‐8) for β_1_ and β_3_. Respectively, with the black dots indicating both parameters were genome-wide significant. The pink and light blue dots indicate variants that were significant after correcting for 1000 tests (p < .05/1000, or p < 5e‐5) for either β_1_ and β_3_. respectively. The purple dots indicate variants where both β_1_ and β_3_ were significant after the multiple testing correction. Finally, the grey dots indication variants with p‐values that failed to meet the multiple testing threshold.

As complex traits are the function of myriad genetic variants, it is necessary to extend the simulation to a polygenic system with relatively small effect sizes. When examining the GxE GWAS results, it becomes immediately clear that, even with 1,000 SNPs and 100,000 observations (from the DZ twins), statistical power is an issue. Specifically, despite simulating a main effect and interaction for every SNP ( β _1_ = β _3_ = $\sqrt{1/1000}$ = 0.0316), only 12 SNPs had genome-wide significant main effects and 4 SNPs had genome-wide significant interaction effects at the genome-wide threshold (p < 5e-8). At a more relaxed multiple testing threshold (p < 0.05/1,000) the numbers jumped to 329 significant main effects and 88 significant interaction effects. A scatterplot of the estimates for the main and interaction effects (Figure 4a) emphasizes that the significant main effects simultaneously overestimate the simulated main effect and underestimate the simulated interaction effect, and *vice versa* for the interaction effects. This is consistent with both a winner’s curse (Xiao and Boehnke, 2009) and a compensatory estimation trade-off between main and interaction effects. While it may seem strange that simulated parameters are not significant, it reflects the reality of genetic associations.

Using only the first 2000 MZ and 2000 DZ twin pairs, the twin analysis easily identifies GxE in the polygenic GxE simulation. Interestingly, the pattern of results is almost identical to the single variant GxE simulation (Figure 3c). Like the single variant simulation, in the polygenic simulation Va accounts for approximately half of the phenotypic variance (Va ≈ 0.5, Vt ≈ 1) in the absence of the moderator and approximately 80% of the phenotypic variance (Va ≈ 2, Vt ≈ 2.5) in the presence of the moderator.

Like the twin model, the PRSxE analyses for the polygenic GxE simulation are consistent with the single variant GxE simulation, with a few notable differences (third panel of Table 2). Both PRS_main_ and PRS_int_ are highly significant when the scores are generated from the GxE discovery model, but the interaction effect PRS is somewhat underestimated. When scores are derived from the standard GWAS summary statistics, we detect significant associations for both the PRS_stand_ as well as the interaction between the PRS_stand_ and the moderator.

### 3.4 Study 4: polygenic model with random normal main effects and interactions

Up to this point, the simulations focused on models where all the variants are associated with the phenotype with the same main and interaction effects. These simulations illustrate the link between GWAS, PRS and twin GxE models by holding the total genetic and interaction variance constant. They also lay the foundation for more complex models of polygenic moderation. In this simulation, the effect sizes for the main and interaction effects are drawn from a normal distribution with a mean of zero and a sd of $\sqrt{1/1000}$ . Accordingly, while many main and/or interaction effects are effectively null, some are positive, others negative with possible effect sizes substantially larger than in study 3.

Starting with the GxE GWAS analyses for 100,000 DZ twin 1 observations, we now see 218 main effects and 106 interaction effects that are genome-wide significant, and an additional 158 main effects and 132 interaction effects that survive a multiple testing correction of α< 0.05/1000 . While these values may be larger than common effect sizes when compared to some complex polygenic traits, the results highlight the fact that when the GxE GWAS effect sizes are large, they can be easily identified (Figure 4b). As is true for moderated regression more broadly, main effects are more likely to be detected than interaction effects. This simple power discrepancy contributes to the underestimation of the prevalence of GxE and the importance of GxE for complex traits.

As with the previous simulations, the twin analysis of 2000 MZ and 2000 DZ twin families easily detects significant GxE (Figure 3d). Further, because the distributions of the main and interaction effects are independent, moderation can amplify or suppress the main effects. Nevertheless, the increase in the total variance in the simulated phenotype in the presence of the moderator is almost entirely due to the increase in genetic variation.

In study 4, the PRSxE analyses using the GxE GWAS vs. the standard GWAS as the discovery samples diverge notably (right panels of Table 2). Specifically, in analyses based on the GxE discovery sample both PRS_main_ and PRS_int_ predict the outcome at the simulated levels. For the PRSxE model using the standard GWAS as the discovery sample, PRS_stand_ is highly predictive of the outcome, but the interaction between PRS_stand_ and the moderator is trivial and not statistically significant. As there are a reasonably large number of variants with genome-wide significant GxE, the lack of an interaction signal when using the standard GWAS model as the discovery sample is unlikely. Instead, the approximately equal mixture of positive and negative interaction effects cancel out when standard PRS methods are used in the PRSxE model.

### 3.5 Ad hoc exploration of the underestimation of the PRS effects

In developing the simulations studies, particularly when smaller sample sizes were used, an interesting pattern emerged where we observed a consistent underestimation of the PRS effect sizes as polygenicity increased. This effect was particularly pronounced for the analyses based on the GxE GWAS discovery analyses, as can be seen in Study 3. Specifically, the PRS effect sizes in all the simulations were generated to be approximately 1. However, when relatively small sample sizes were used, the estimated PRS effects were substantially smaller than 1. As statistical power in PRS analyses depends on the precision of the discovery sample estimates (Dudbridge, 2013; Palla and Dudbridge, 2015), both the magnitude of the effects and the size of the discovery sample play a pivotal role in generating accurate PRS in the target sample. Accordingly, it is likely that the underestimated PRS effects stem from the lack of precision in the estimation of the respective coefficients in the discovery analyses, with the interaction parameters being less precise than main effects. Interestingly, the downward biased PRS effect resulted in inflated intercept and the moderator coefficients (that deviate from the simulated values of 0). Essentially, the overestimation of these effects allowed the models to compensate for the underestimation of the PRSs.

**FIGURE 5:**
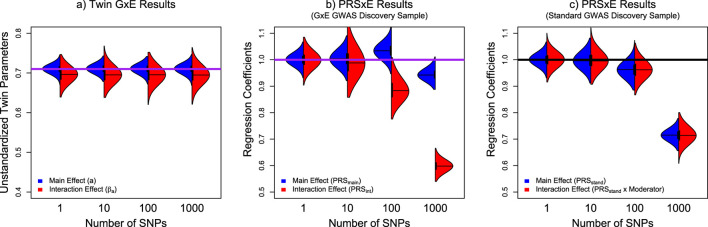
Violin plots of the distributions of the respective main effect and interaction effect parameters from the Twin GxE model, PRSs derived from the GxE GWAS discovery analyses, and a PRS derived from standard GWAS methods. Note: Panel **(a)** presents the distribution of additive genetic main effect (a) in blue and interaction effects ( β _a_) in red as described in Figure 2. Panel **(b)** presents the distribution of the coefficients for PRS_main_ in red and PRS_int_ in blue as described in Equations 3–5. Panel **(c)** presents the distribution of the coefficients for PRS_stand_ in red and the interaction between PRS_stand_ and the moderator in blue as described in Equations 6, 7. The solid purple line indicates the simulated values for the relevant parameters.

To investigate the cause of the underestimated PRS effects, we conducted a follow-up analysis where we varied the number of simulated SNPs (N_snps_ = 1, 10, 100, 1,000), repeating the simulation 1,000 times for each condition with a sample size at 20,000. Thus, the 1 and 10 SNP conditions will be overpowered, but the 100 and 1,000 SNP conditions will be underpowered. As the analyses become increasingly underpowered, we should observe an increasing downward bias in the PRS estimates. As the twin parameters are sufficiently powered across all these conditions, the twin model serves as a positive control. Specifically, the additive genetic main and interaction parameters in the twin model should be approximately 0.71, and the PRSxE main and interaction parameters for both discovery samples should be 1. As shown in the left panel of Figure 5, the twin model estimates are close to the simulated parameters, with a very slight but consistent underestimation of the interaction parameter due to the inclusion of common and unique environmental moderation (see Verhulst et al., 2019 for a more complete explanation of this bias). In both PRSxE models, as the polygenicity increased, so did the underestimation. However, in models using the GxE GWAS as the discovery sample, PRS_int_ is substantially more affected than PRS_main_. Alternatively, when using the standard GWAS as the discovery sample, because there is only PRS_stand_, the main effect and interaction effect underestimation is symmetrical. These results suggest that PRSs derived from underpowered discovery samples will underestimate the polygenic effect.

### 3.6 Statistical power comparisons for GWAS main and interaction effects

Since statistical power is a constant concern in GWAS, and particularly GxE GWAS, Monte Carlo power calculations were conducted to identify the approximate sample size necessary to achieve 80% power to detect genetic associations at the genome-wide significance level for four main and interaction effect sizes ( β _1_ = β _3_ = {1.0, 0.316, 0.1, 0.0316}; Table 4). The power analyses highlight the extreme discrepancy in power between the β _1_ and β _3_ coefficients. Specifically, when the values of β _1_ and β _3_, are equal, it takes more than twice as many observations to achieve 80% power for β _3_ as compared with β _1_, even though both parameters are estimated in the same model. Put another way, if the interaction effect size is the same as the main effect size, if an analysis has 80% power to detect the main effect, it likely has around 15% power to detect the interaction effect. Alternatively, if an analysis has 80% power to detect the interaction effect, it has almost perfect power to detect the main effect. Importantly, while β values greater than 0.1 are unrealistic for most complex traits, effect sizes in the 0.05 range are reasonable. Thus, it may take approximately 500,000 observations to achieve 80% power to detect significant β _3_ coefficients for many complex traits. Importantly, when the sample size in the discovery sample has 80% power to detect the association, the underestimation of both the main and interaction effects are ameliorated.

**TABLE 4:** Power analysis results comparing the sample size and statistical power to detect main effects and interaction effects in moderated regression analyses.

Number of SNPs	Effect size	Sample size for 80% power for the main effect	Power to detect the interaction effect from the main effect power analysis	Sample size for 80% power for the interaction effect	Power to detect the main effect based on the interaction effect sample size
1	1.0	84	0.23	159	1.00
10	0.316	2,340	0.14	4,950	1.00
100	0.10	24,744	0.13	52,529	1.00
1,000	0.0316	251,674	0.13	528,683	1.00

The effect size was determined as $\sqrt{1/N_{SNPs}}$ to remain consistent with the simulation studies. The sample size for 80% power for the main and interaction effects was identified via Monte Carlo simulations and should be considered approximate. The power to detect the interaction effect (or the main effect) was based on the sample size required to achieve 80% power from the main effect (interaction effect) analysis.

## 4 Discussion

The simulation studies demonstrate substantial consistency in the ability to detect GxE across analytical approaches. The twin analyses identified increasing phenotypic heritability at riskier levels of the moderating environment. The PRSxE analyses identified both genetic main and interaction effects. The GxE GWASs identified interactions with individual genetic variants. Unsurprisingly, as we increased the polygenicity of the main and interaction effects (thereby shrinking their effect sizes), the power to detect genome-wide associations and interactions for individual variants declines. Nevertheless, both twin and PRS analyses were able to identify robust main and moderated effects with reasonably small sample sizes (i.e., 2,000 MZ and DZ twin pairs and 5,000 individuals in the PRS target sample). Therefore, twin and PRSxE analyses can be used to supplement investigations by focusing attention on (or eliminating) phenotype-moderator combinations that likely exhibit GxE.

Interestingly, while twin methods have struggled to remain relevant in the genomic era, standard twin GxE methods were extremely robust to variation in polygenicity and the direction of the interactions at the individual variant levels. For simulation studies 2 and 3, the pattern of GxE in the twin models was the same regardless of the number of SNPs included in the data generating process. Further, the twin model effectively identified GxE even when the PRSxE models underestimated the effects or failed to identify moderation. Accordingly, twin GxE methods remain robust to many of the limitations that affect genome-wide approaches.

Across the simulation studies, it is possible to broadly conclude that the PRSxE models accurately recover the simulated values for both the main and interaction effects. However, there are three notable caveats to this conclusion. First, the pattern of individual variant GxE has a substantial impact on the PRSxE moderation effects. If the sign of the moderation was consistent across variants (e.g., the interaction effects consistently amplified the genetic associations), the PRS derived from standard GWAS summary statistics performed slightly better than PRSs derived from GxE GWAS summary statistics. However, if some interaction coefficients were positive and others negative, constructing the PRSs from standard GWAS regression weights led to the conclusion that GxE did not exist. When using PRSs derived from the GxE GWAS discovery analysis, we identified significant moderation. Effectively, using standard GWAS summary statistics to construct PRSs in a PRSxE models assumes that every SNP interacts with the environment proportional to the magnitude and direction of the discovery GWAS. If GxE amplifies the associations for some loci and suppresses others, aggregating genetic effects using standard PRS methods will effectively cancel out any moderation. Because standard GWASs do not include moderation, it is possible that using standard GWAS summary statistics to generate PRSs may lead to the conclusion that GxE does not exist, when, in fact, it does.

Second, as we demonstrate in the *post hoc* analyses, the sample size of the discovery analysis plays a critical role for identifying polygenic signatures. While this has been pointed out for linear GWASs (Dudbridge, 2013; Palla and Dudbridge, 2015), it is worth repeating in the context of GxE. If the discovery analyses are not sufficiently powered to detect the genetic associations, the regression weights will contain substantial levels of stochastic noise, and the PRSxE analyses will underestimate the genetic signal for both main and interaction effects. As interaction effects require larger samples to achieve the same level of statistical power, the underestimation of the interaction PRSs was more pronounced. Across the simulation studies, the genetic variance was held constant to enhance the comparability of the results. In real analyses every effect size is different. Therefore, in real analyses PRSs will necessarily be constructed from discovery GWASs that have a mixture of effect sizes, with some associations being sufficiently powered and others underpowered. Accordingly, to the extent to which underpowered variants are included in a PRS, the results likely underestimate the importance of the genetic factors for the phenotype.

Finally, the accuracy of the *r* ^2^ statistic, which indexes the proportion of variation accounted for by the predictors in the full model, does not reflect the *r* ^2^ for specific levels of the moderator. In the single variant GxE model, the model *r* ^2^ was substantially overestimated when the moderator was absent and underestimated when it was present. In polygenic models, aggregating *r* ^2^ across a mixture of over- and underestimation in real samples may leave PRSs disconnected from any specific level of the moderator. Thus, while *r* ^2^ may be an effective metric for standard PRS analyses, its usefulness for PRSxE analyses may be limited.

Importantly, with the proliferation of high-quality, publicly accessible GWAS summary statistics, PRS and PRSxE methods are entering a phase of rapid methodological development (Ni et al., 2021). For example, methods such as PRS-CSx (Ruan et al., 2022), MegaPRS (Zhang et al., 2021), LDPred2 (Privé et al., 2021), and SBayesR (Lloyd-Jones et al., 2019) extend PRS analyses to diverse ancestry groups, re-weight the finding by including functional genomic information, and utilize powerful Bayesian methods. While we do not use these advanced PRS methods in our simulation studies, our results nevertheless can inform analyses that do. Furthermore, while our simulations do not include covariates, Type 1 Error rates can be better controlled by including higher order polynomials of the covariates (Jayasinghe et al., 2024). Thus, our findings add to the broader perspective on the accuracy of PRSs for complex traits.

The GxE GWAS simulations generally confirmed the existing knowledge regarding the low levels of statistical power and the corresponding need to have very large sample sizes to identify GxE for individual variants. However, connecting GWAS data on GxE for individual variants to twin and PRS data challenges the perception that the underlying genomic mechanisms for twin and molecular methods are categorically distinct. While twin, PRS, and GWAS methods use different data types, rely on different statistical assumptions, and conceptualize “genes” in different ways, they can all be reduced to the same basic biological principals. Individual variant analyses, like GxE GWASs, promise extremely precise levels of biological specificity, which informs the etiology of complex traits and can be leveraged to advance personalized, genomic medicine. It will take an enormous amount of time, money, and effort to collect hundreds of thousands (and likely millions) of high-quality genotypes and phenotypes, not to mention numerous potential moderators for each phenotype. Accordingly, it is essential to use data and methods, that only require a few thousand observations, to prioritize the phenotype-moderator combinations with the highest chances of illuminating GxE for individual variants.

One of the strengths of our simulation studies is the flexibility to generate data that simultaneously satisfies the requirements of twin, GWAS and PRS analyses. However, such data is only realistically obtained within a simulation setting, as each method has substantially different requirements. Moreover, despite being able to detect GxE with each method, the research questions that can be answered with each analytical approach vary substantially. We discuss the key considerations for each method below and summarize them in Table 5.

**TABLE 5:** Summary of the hypotheses, strengths and limitations of GxE analyses using twin, PRS and GWAS methods.

GxE method	Sample requirements	GxE Hypothesis	Strengths	Limitations
Twin	MZ and DZ Twins	Does the proportion of genetic variance differ based on the level of the moderator?	• Robust to most differences in the genetic architecture of the phenotype and the pattern of GxE for individual variants • Well-validated and accepted methods with known strengths and limitations	• Twins are a relatively rare populations and require have unique recruitment barriers • Latent variables have for low levels of biological specificity making translation difficult
PRS	Discovery Sample and Target Sample	Does the PRS have different effects based on the level of the moderator?	• Data can be contributed to future GWASs and GxE GWAS meta-analyses • It is possible to stratify individual risk in ways that could be used in clinical settings (for some phenotypes) • If an appropriate discovery sample is identified, PRS target samples can be relatively small	• Requires two samples • Phenotypic and moderator heterogeneity across samples may not be assumed • Sensitive to the pattern of GxE for individual variants • GxE GWASs are rare and may not be available for the phenotype-moderator combinations of interest
GWAS	Single Sample	Do individual variants have different effects based on the level of the moderator?	• Very high levels of biological specificity • Direct extensions drug development and treatment	• Sample size or statistical power

Twin studies focus on how measured environmental moderators amplify or suppress the additive genetic, common and unique environmental variance components. Focusing on the additive genetic variance component, twin GxE models treat “genes” as a latent biological predisposition for a behavior that can be regulated by the environment. The latent biological factor effectively aggregates genome-wide genetic associations into a single variable that does not require DNA collection, instead leveraging the differences between the variances and covariances of MZ and DZ twins (Neale and Cardon, 1992; Purcell, 2002). Because latent variables cannot be measured directly and thus must be inferred from the existence of other measurable variables, twin methods cannot be used to identify actionable biological interventions. Nevertheless, twin models require substantially smaller sample sizes to detect GxE, making them extremely valuable for identifying phenotype-moderator combinations with the potential for clinical relevance.

PRS analyses, and PRSxE studies by extension, aggregate the effect of relevant alleles for a phenotype across the genome (Cross et al., 2022). PRS analyses can effectively test whether genetic factors play a role in a behavior by projecting estimated genetic associations from the GWAS of a large discovery sample into a smaller genetically assayed target sample. PRSxE analyses refine the potential research question by asking whether environmental factors increase or decrease the importance of the genetic signal. As with twin studies, PRS and PRSxE methods cannot directly identify actionable genetic interventions, as they aggregate signals from across the genome. However, PRSs can be used to stratify a person’s genetic risk of developing a disease or other phenotype. By extension, it is possible that PRSxE methods can be used to further personalize the prediction by integrating the genetic signal with an environmental moderator. If conducted appropriately, this integration can be instrumental for enhancing personalized medicine (Verhulst and Benstock, 2023). As most current PRS and PRSxE analyses utilize published GWAS summary statistics as the discovery sample, collecting or identifying data for a target sample is of critical importance. Importantly, sample sizes for these target samples can be substantially smaller than the discovery GWAS. While we used 5,000 observations in our target datasets, with sufficiently powered discovery GWAS it is possible to detect genetic signals with sample sizes in the hundreds (Chang et al., 2019).

Finally, GxE GWAS analyses provide the most biological specificity, by identifying specific SNPs that interact with an environmental moderator. However, the statistical power to detect moderated associations for individual SNPs is low. Accordingly, GxE GWASs with currently available data (including those from biobanks) may only be able to identify GxE with large moderation effects. One method of addressing power concerns in GWAS has been to develop consortia that share data and results, thereby increasing sample sizes (Adams et al., 2025; International Schizophrenia Consortium et al., 2009). Within these consortia, phenotypic heterogeneity has been a major concern for GWAS meta-analyses. Differential assessment and ascertainment strategies can obscure the interpretation of any potential associations (Cai et al., 2025). In GxE meta-analyses, not only is phenotypic heterogeneity a concern, but moderator heterogeneity will introduce additional challenges. For example, many conceptualizations of adverse life events aggregate stressors from numerous categories. While aggregated scales can increase statistical power in many situations, if individual stressors have divergent moderating effects, such effects may inhibit the detection of GxE.

Many of the lessons learned from GWASs extend to genome-wide GxE analyses, but due to the increased complexity of moderated regression over linear regression, slight extensions are required. For example, one of the central lessons from GWASs is the extreme polygenicity of complex traits: hundreds or thousands of independent loci are associated with each behavioral outcome, with individual associations exerting very small effects and thus requiring extremely large sample sizes to detect. Moderated genetic associations will likely be equally polygenic, but as interactions are inherently less powerful, they will require even larger samples. Substantial reductions in genotyping costs over the past 2 decades have allowed for multiple historic efforts aimed at recruiting hundreds of thousands of participants assessed on a wide range of outcomes and moderators (Sudlow et al., 2015; The All of Us Research Program Investigators, 2019). Thus, the justification for eschewing genome-wide GxE due to power concerns is decreasing.

## 5 Conclusion

While GxE plays a critical role in the etiology of many complex traits, as demonstrated by the plethora of GxE research in twins, genome-wide GxE research has fallen behind other genomic investigations. In part, the hesitation to conduct genome-wide GxE research is a function of perceptions of low rates of statistical power and skepticism from controversial findings from the candidate gene-environment interaction literature (Culverhouse et al., 2018; Duncan and Keller, 2011). While reasonable, such perceptions have delayed genome-wide GxE investigations. The methodological consistency demonstrated by the current simulations highlight the importance of leveraging multiple methods for identifying GxE. Thus, the robust twin GxE literature should give researchers a sense of hope that corresponding findings can be identified in genome-wide data.

## Data Availability

The datasets presented in this study can be found in online repositories. The names of the repository/repositories and accession number(s) can be found below: https://github.com/bradverhulst/GxESimulation/. Further inquiries can be directed to the corresponding author.
